# Assembly of Protein Cages for Drug Delivery

**DOI:** 10.3390/pharmaceutics14122609

**Published:** 2022-11-26

**Authors:** Xiaoxuan Yu, Zihui Weng, Ziyang Zhao, Jiayun Xu, Zhenhui Qi, Junqiu Liu

**Affiliations:** 1 Key Laboratory of Organosilicon Chemistry and Material Technology, Ministry of Education, College of Material, Chemistry and Chemical Engineering, Hangzhou Normal University, Hangzhou 311121, China; 2 Sino-German Joint Research Lab for Space Biomaterials and Translational Technology, School of Life Sciences, Northwestern Polytechnical University, Xi’an 710072, China

**Keywords:** protein cage, nanoparticles, drug delivery, VLPs

## Abstract

Nanoparticles (NPs) have been widely used as target delivery vehicles for therapeutic goods; however, compared with inorganic and organic nanomaterials, protein nanomaterials have better biocompatibility and can self-assemble into highly ordered cage-like structures, which are more favorable for applications in targeted drug delivery. In this review, we concentrate on the typical protein cage nanoparticles drugs encapsulation processes, such as drug fusion expression, diffusion, electrostatic contact, covalent binding, and protein cage disassembly/recombination. The usage of protein cage nanoparticles in biomedicine is also briefly discussed. These materials can be utilized to transport small molecules, peptides, siRNA, and other medications for anti-tumor, contrast, etc.

## 1. Introduction

**Figure 1:**
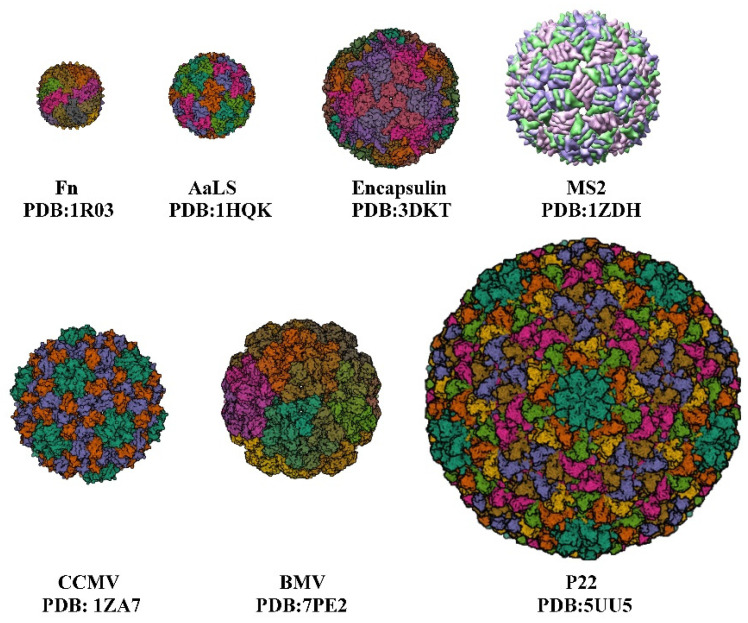
Several protein cages of different sizes. Fn, Ferritin cage; AaLS, *Aquifex aeolicus*; encapsulin protein cage; MS2; CCMV, Cowpea chlorotic mottle virus; BMV, Bromemosaic virus; P22, Bacteriophage.

The development of nanocarriers for drug delivery has been of great interest to many researchers over recent decades, as they overcome the limitations of traditional drug delivery methods [1]. Nanoparticles (NPs) have unique properties in terms of their physical, optical, chemical, and therapeutic properties. Traditional drug delivery carriers, such as non-nanomedicines and inorganic NPs, face high toxicity and low delivery efficiency, making naturally derived nanocarriers viable alternatives. Protein nanocages [2], such as viruses, virus-like particles, and ferritin, are formed by the self-assembly of protein subunits to form cage-like structures, and they consist of varying numbers of subunits to form protein cages of varying sizes, with cage diameters ranging from a few nanometers to tens of nanometers (Figure 1, Table 1), which are suitable for the encapsulation and application of various drugs, respectively. Virus-like particles (VLPs) are a class of multimeric proteins derived from viral capsids that self-assemble into protein cage NPs with hollow interiors. The creation and structure of VLPs are identical to those of wild-type viruses, but since they lack the viral genome, they are not harmful. The virus’s shell is a symmetrical supramolecular complex composed of proteins. Each icosahedral virus contains 12 pentameric substructures that are located according to their triangulation number (T). When the subunits remain the same size, increasing the number of subunits will increase the T number and the size of the capsid [3]. Among protein NPs, VLPs are the most advantageous. In terms of biocompatibility, cellular absorption efficiency, and stability in PBS buffers, cell culture media, and mouse serum [4,5,6], VLPs surpass conventional organic or inorganic NPs. Targeted peptide-modified VLPs that are primarily localized in tumor tissues can prevent the dissemination of DOX to multiple organs, hence reducing therapy side effects [7]. VLPs can also reduce cytotoxicity from encapsulated toxic cargoes [8].

Today, researchers are paying more attention to the functionalization of protein cage NPs. It can be flexibly designed by protein engineering, and the inner and outer surfaces of protein cage NPs can be altered in a variety of ways, including affinity tags, antibodies, particular amino acids (e.g., cysteine), and targeting peptides to carry out particular functions of various purposes [9]. The main topic of this presentation will be the utilization of protein cage NPs as drug carriers to encapsulate pharmaceuticals. Additionally, it describes how protein cages are used to encapsulate tiny molecules, nucleic acids, peptides, and proteins for imaging, cancer therapy, delivering nucleic acids, and many other purposes. These methods will serve as a foundation for the later creation of novel protein cages.

**Table 1:** Several triangulations and external/inner diameters of protein cage NPs.

Protein Cage NPs Type	T ^1^	External/Inner Diameter	Number of Identical Subunits	References
Fn	-	12 nm/8 nm	24	[10]
AaLS	1	15.4 nm/9 nm	60	[11]
Encapsulin	1	24 nm/20 nm	60	[12]
P22	7	PC 59.6 nm/44.6 nm EX/WB 64.8 nm/51.6 nm	420	[13]
CCMV	3	28 nm/18 nm	180	[14,15]
MS2	3	27 nm/-	180	[16]
BMV	3	28 nm/-	180	[17]

^1^ Triangulation number.

## 2. Strategies for Drugs Encapsulation in Various Protein Cage Nanoparticles

Due to their good biocompatibility, protein cage NPs are commonly used to encapsulate drugs and enzymes. Protein cage NP encapsulation generally uses the following methods:

(1) Diffusion. It is only possible to load a limited amount of small molecule cargoes (<5 Å) or metal ions smaller than the pores of the protein cage NPs. Additionally, some protein cage NPs allow drugs to enter the interior of the protein cage NPs by thermally responding to cargo pores. (2) Electrostatic adsorption. It facilitates the selective adsorption of oppositely charged drugs by charged protein cage NPs. (3) Covalent modification. Enhanced encapsulation efficiency of drugs can be achieved by chemical modification or specific short peptide modification. (4) Disassembly/reassembly. Reversible disassembly or reorganization of protein cage NPs by adjusting pH or salt concentration. (5) Fusion expression. The fusion and expression of encapsulated drugs on protein cages, by combining drugs with scaffold proteins and encapsulating them in cages, can increase the local concentration of the reaction. 

### 2.1. Diffusion

**Figure 2:**
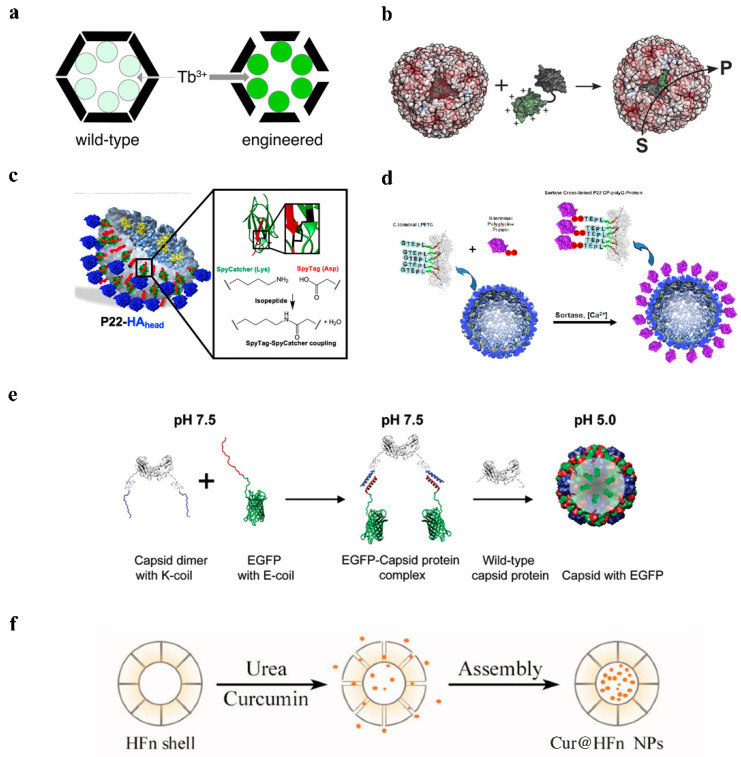
The strategy of encapsulating drugs with protein cage NPs. (**a**) TB^3+^ diffuses into encapsulin. Reprinted with permission from Ref. [19]. Copyright 2018, American Chemical Society. (**b**) Positively charged green fluorescent protein GFP (+36) could be encapsulated into AfFtn. Reprinted with permission from Ref. [21]. Copyright 2021, Wiley-VCH Verlag GmbH & Co. KGaA, Weinheim. (**c**) SC/ST create a covalent isopeptide bond to encapsulate HA_head_. Reprinted with permission from Ref. [32]. Copyright 2020, American Chemical Society. (**d**) To encapsulate HA_head_, LPETG amino acid sequences and polyglycine establish a covalent connection. Reprinted with permission from Ref. [33]. Copyright 2017, American Chemical Society. (**e**) EGFP encapsulation into CCMV VLPs under pH control. Reprinted with permission from Ref. [34]. Copyright 2009, American Chemical Society. (**f**) Urea-regulated HFn disassembly/reassembly. Reprinted with permission from Ref. [35]. Copyright 2022, Informa Healthcare.

Passive diffusion allows drugs to be loaded into Human H ferritin (HFn) through protein pores. The drugs are packaged into the cage-like structure only after passing through the pores. Although there are fewer drugs loaded into the HFn nanocages, the packaging efficiency is low, and they are limited to some small molecule drugs (with size <5 Å) or some metal ions. Thus, a natural temperature-sensitive thermal-response channel on HFn shell has been discovered, facilitating drug entry into protein cages [6]. A ferritin cage with a 4-fold channel was formed by artificially removing the E-helix formed by the C-terminal channel. To ensure the stability of the protein cage NPs, Nicked-HF partially forms a quadruple channel, and the drug is actively encapsulated into the cage-like structure via this channel [18]. Similarly, the natural encapsulins have pores of 3–4 Å in diameter, which are only suitable for some metal ions to pass through. With the pores modified to 11 Å, Tb^3+^ is able to diffuse into the cage and the transport rate is increased 7-fold, making it easier for encapsulins to transport drugs [19] (Figure 2a). The passive diffusion of drugs has certain limitations, and drugs can be lost during transport.

### 2.2. Electrostatic Adsorption

The use of electrostatic charge-mediated interactions facilitated the selective encapsulation of negatively/positively charged drugs by positively/negatively charged protein cage NPs. *Escherichia coli* alkaline phosphatase (PhoA) can be encapsulated by the positively charged protein capsid MS2, and the negatively charged acidic peptide can be fused to PhoA, which can further improve the encapsulation efficiency [20]. Negatively charged siRNA can interact electrostatically with the arginine-binding domain of the CCMV [14]. *Archaeoglobus fulgidus* ferritin (AfFtn) has negatively charged proteins inside, which can encapsulate positively charged gold nanoparticles [21] (Figure 2b). In addition, positively charged green fluorescent protein GFP (+36) was used to confirm that positively charged particles could be encapsulated into AfFtn [22]. Layer-by-layer, the functional protein cage NPs formed by adding oppositely charged macromolecules to the surface will have greater application opportunities [23].

### 2.3. Covalent Modification

The two main types of covalent modifications used to encapsulate drugs are covalent modifications using natural and non-natural amino acid residues [24], and protein-mediated covalent modifications [25].

#### 2.3.1. Covalent Modification of Natural and Non-Natural Amino Acid Residues

The side chains of canonical amino acids are reactive, meaning they have the ability to form biocompatible covalent bonds [24]. These amino acids include cysteine, lysine, glutamic acid, aspartic acid, and tyrosine. Cysteine covalent crosslinking relies on the free sulfhydryl group on the cysteine, is chemically loaded onto protein cage NPs by thiol-maleimide Michael-type addition, and can occur at any desired location on the protein surface, e.g., inside or outside the protein cage NPs. There are more established synthetic protocols for maleimide-linked drugs, which are relatively easy to obtain, so cysteine covalent links are commonly used. Aldoxorubicin (AlDox) is a commonly used anticancer prodrug that encapsulates the drug through cysteine residues-maleimide Michael-type addition to 123-site Cys located on the outer surface of encapsulin protein cage NPs, and the activity of AlDox is not reduced by a covalent modification to the protein cage [12]. 

AaLS isolated from the hyperthermophile *Aquifex aeolicus* has been genetically modified to contain cysteine at position 108, which has been used to encapsulate drugs such as AlDox and bortezomib [11]. The Gd (III)-DOTA-Mal complexes were also successfully linked to the cysteine residue at position 108 of the AaLS protein cage [26]. Selenocysteine (Sec) is oxidized to selenocysteine dimer (Sec2) in air. Using AaLS mutants with one cysteine, Sec is encapsulated at sequence position 122 of each subunit of AaLS, and Sec2 effectively forms a Sec sulfide linked coupling with AaLS without altering the capsid’s original structure [27]. Using reducing agents such as glutathione or dithiothreitol, the encapsulated Sec can be quantitatively released from AaLS–ICSec [27]. Ferritin-based nanocarriers with 20 nm diameter utilize internally mutated cysteine residues to encapsulate SOD [28]. In addition, *Salmonella typhimurium* bacteriophage P22 VLPs cysteine mutants (S133C or K118C) were also used to encapsulate AlDox [29].

Lysine is mostly exposed on the surface of protein cage NPs and can be used to link small molecule drugs, and the primary amine (R-NH_2_) on lysine reacts with N-hydroxysuccinimidyl esters (NHS-esters). The carboxyl groups of aspartic acid and glutamic acid can react with primary amines in the presence of carbodiimide. The phenol group of tyrosine can be coupled and the tyrosine can be interlinked with other tyrosine [30]. This method of mutating specific amino acids on the protein cage NPs also has limitations, potentially leading to changes in the original structure of the protein cage NPs or a reduction in the structural stability of the protein cage NPs.

#### 2.3.2. Protein-Mediated Encapsulation Strategies

Spytag (ST)/Spycatcher (SC) covalent attachment strategy: The protein cage NP’s structure is unaffected by ST fusion expression outside of the protein cage NPs, and SC can target to identify ST and create covalent isopeptide bonds [31]. After modifying SC on the drug, the drug can be encapsulated onto protein cage NPs; protein cage NPs have the same formation process and structure as wild viruses, but they are not infectious and can be used for vaccine production. Seasonal influenza A virus (IAV) often undergoes gene mutations or gene recombination, resulting in the need for frequent updates of IAV and the use of P22 VLPs as a vector for constructing vaccines, which have different globular head structural domains of hemagglutinin (HA) proteins, allowing for rapid updates of IAV. Expression of SP at the C-terminus of the CP of P22 VLPs allows ST to be expressed outside of VLPs and ST/SC to bind covalently, resulting in a P22-HA_head_ that activates antigen-specific immune responses in mice (Figure 2c), and exhibiting enhanced immunogenicity and potential for antigen delivery [29,32]. AaLS also uses the irreversible covalent isopeptide bond formed by ST/SC to modify the epidermal growth factor receptor (EGFR) to the outside of the protein cage, and each AaLS nanoparticle successfully attaches to an average of approximately 30 TRAIL and EGFRAfb molecules [25].

Sortase-mediated ligation strategy: Sortases catalyze the formation of peptide bonds between the threonine of proteins containing LPETG amino acid sequences at the C-terminus, and the glycine of proteins containing polyglycine sequences at the N-terminus [33]. The fusion expression of a sequence LPETG at the C-terminus of the coat protein of P22 VLPs, modified with polyglycine at the N-terminal end of the drug, similarly allows stable loading of different drugs onto protein cage NPs, and modifications on the outer side of the protein cage NPs that facilitate the encapsulation of some large protein or protein structural domains [33] (Figure 2d). The covalent bonds formed after chemical modification require the participation of organic solvents which, to a certain extent, can cause damage to the proteins and is detrimental to the encapsulation of the drug, while the covalent bonds formed by using peptides or proteins can be done in a mild buffer system, which can reduce the loss of activity to the biological material [33].

### 2.4. Disassembly/Reassembly

Certain protein cages have the capacity to regulate the breakdown and reassembly of cage-like structures under particular circumstances. For instance, ferritin may be collected from plants, animals, and microbes and is an intracellular spherical protein that stores iron. To load pharmaceuticals into the ferritin cage’s cavities, the protein can be automatically formed into cage-like structures with cavities, and the breakdown and reassembly of the protein cage can be regulated by the pH value and urea concentration [36]. Additionally, the pH value can control the capsid structure of the cowpea chlorotic mottle virus (CCMV).

1. pH-regulated drug encapsulation strategy for protein cages

The ferritin cage can be dissociated in phosphate buffer with a pH of 2.5, and then adriamycin is added. The protein shell is put back together, and the drug is successfully encapsulated in the protein cage after the pH is raised to 7.4 using NaOH (Figure 2e); however, if the pH of the solution is directly raised from 2.5 to 7.4, drug and protein aggregation will occur, having irreversible effects on proteins. The drug encapsulation of pH-regulated ferritin cages was subsequently enhanced, and it was discovered that adriamycin was more soluble and stable at pH 3–4. Additionally, pH from 2.4 to 4 may be more advantageous for ferritin synthesis than pH from 2.4 to 7.4, and glycine-acetate buffer (pH 2.5) was also discovered to have greater protein recovery. Due to the improvement of the procedure for disassembling and reconstituting pH-regulated ferritin cages, the ferritin cages were broken down in glycine-acetate buffer (pH 2.5) and then adriamycin was added to encourage the creation of ferritin cages at a pH of 4. After aspirating the free medication, the buffer was changed to 7.4 before continuing. One ferritin cage can contain up to 28 adriamycin molecules thanks to this mechanism’s ability to restore ferritin cage complexes and increase the drug-loading capacity of ferritin cages [5,35].

Drugs can be encapsulated in the interior of the capsid of the CCMV. The capsid was disassembled into 90 capsid protein dimers at pH 7.5, and when the pH was reduced to 5.0, the capsid structure reorganized. This reversible disassembly and reorganization were employed to encapsulate a variety of medicines [37].

2. Urea-regulated drug encapsulation strategy for protein cages

PH-regulated drug encapsulation techniques for ferritin cages have been improved; however, a recent study has demonstrated that acidic pH breakdown produces irreparable holes in the surface of ferritin cages, leading more individuals to opt for the milder urea to manage ferritin cage disassembly and reconstitution [38] (Figure 2f). Complete dissociation of HFn in 8 M urea was followed by the addition of the drug, which was attached to ferritin. The gradient removed the urea, and the assembly was then put back together to create a ferritin cage encapsulating the medication [39].

### 2.5. Fusion Expression

The use of protein nanocages to load peptide drugs, thereby encapsulating drugs in a purely biological manner, can successfully avoid the loss of activity caused by chemical reagents used in the encapsulation process. Loading protein drugs, such as peptides or enzymes, into the interior of protein nanocages can also protect the drugs from denaturation and degradation, improving the drug’s stability and effectiveness.

#### 2.5.1. P22 VLPs

P22 VLPs are guided by scaffolding protein (SP) to assemble 420 coat protein (CP) into cage-like structures, and there are two commonly used methods for expressing drug fusions on the P22 protein cage [40].

**Figure 3:**
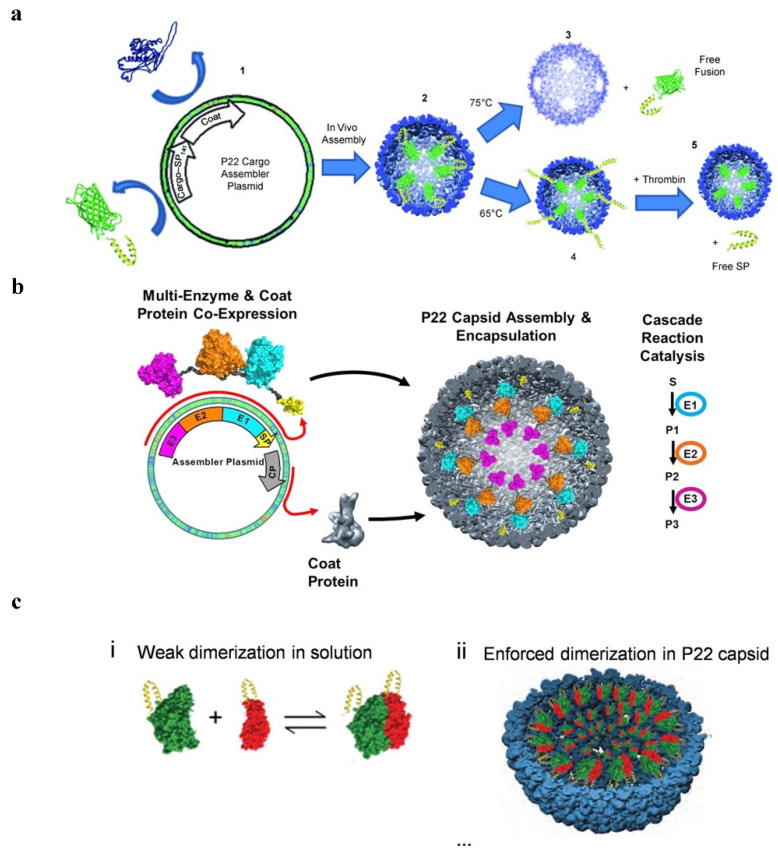
The strategy of drug gusion expression on SP of P22 VLPs. (**a**) P22 self-assembly with coat protein and cargo-scaffold-protein fusion. Reprinted with permission from Ref. [51]. Copyright 2011, WILEY-VCH Verlag GmbH & Co. KGaA, Weinheim. (**b**) Continuous expression of P22 VLPs with three enzymes under the control of the same promoter. Reprinted with permission from Ref. [44]. Copyright 2014, American Chemical Society. (**c**) Two plasmids separately containing the hydrogenase cargo (hyaA-SP and hyaB-SP) and coat protein (CP) under the control of different promoters, to produce P22 capsids packed with both subunits of EcHyd-1. Reprinted with permission from Ref. [42]. Copyright 2022, American Chemical Society.

1. Using an SP fusion technique that does not interfere with the self-assembly of the SP-guided CP, one capable of encasing the target protein within the P22 capsid, and fusing the target gene to the N-terminal end of a shortened SP [41]. It can be used, for instance, to encapsulate certain peptide medications and enzymes. Such encapsulation can enhance the local concentration of the drug protein within a specified range, and the local concentration of the drug protein can reach more than 300 mg/mL while still maintaining the original action of the drug [42]. There are several common strategies for drug fusion expression on SP of P22 VLPs:

(1) Fusion at the N-terminal end of one SP to express a target gene, enabling the medication to be contained inside the P22 VLPs [43] (Figure 3a). (2) Simultaneous production of two or more target proteins at one SP’s N-terminus. Directly existing target proteins can also occur in a cascade reaction [44] (Figure 3b). (3) Two separate target genes are expressed through fusion at the N-terminal ends of two different SPs, causing them to co-assemble in a protein cage [45] (Figure 3c). It has recently been reported in the literature that an in vitro assembly method for cargo encapsulation in P22 VLPs has been designed which can control the amount of cargo encapsulation. By adjusting the ratio of encapsulated cargo to wild-type SP, this method can quantitatively encapsulate drugs into protein cages, and wild-type SP and misfolded proteins can escape the cages [46].

2. Using a CP fusion technique, each VLP can hold a specific quantity of medicines that are genetically linked to the CP of P22 VLPs (420 drugs). For instance, the N-terminal fusion of P22 CP expresses peptide drugs, but the peptide drugs are encapsulated inside the protein cage, and the histone proteinase b is attached to the drugs. Cathepsin B (CTB) is highly expressed in tumor cells, and it can regulate the release of peptide drugs in the tumor site [47].

#### 2.5.2. Ferritin Double-Chambered Nanocage (DCNC)

The ferritin double-chambered nanocage (DCNC) is made up of a double chambered short-length ferritin (sFt) construct that has a fusion at the N-terminal end that should be multivalent clot-targeting peptides (CLT), and a big protein microplasmin (μPn) at the C-terminal end. CLT target fibrin–fibronectin complexes in clots, and μPn effectively dissolves clots in thrombi and can target DCNC to tumor sites to improve intra-tumor drug delivery [48,49].

#### 2.5.3. AaLS

Recently, AaLS nanocages have also been used to deliver peptide drugs. The fusion of periostin peptide (PP) to the C-terminus of the AaLS subunit allows drug expression on the surface of AaLS nanocages, and the pro-angiogenic activity of AaLS-PP was detected to be higher than that of free PP. Therefore, AaLS can be a good carrier for transporting peptide drugs [50].

## 3. Application of Protein Cage Nanoparticles

An ideal nanomedicine carrier should be able to target specific cells, provide large concentrations of therapeutic drugs, have remarkable physical and chemical properties, be biocompatible, and be easy to disintegrate in vivo [36,52].

This makes protein cage NPs the ideal delivery mechanism for peptides, proteins, nucleic acids, and small molecules. In this article, we discuss the use of protein cage NPs for targeting to various tumor areas for tumor therapy, siRNA administration, peptide and protein drug delivery, and contrast techniques. We have investigated numerous encapsulation methodologies for protein cage NPs vectors.

### 3.1. Anti-Tumor

Targeted peptides are frequently utilized in the design of antitumor medications because highly efficient antitumor drugs should be directed specifically to tumor cells and delivered into tumor tissue to destroy tumor cells and avoid damaging normal cells. Protein cage NPs are protein-based nanocages, and by utilizing genetic engineering, it is quite simple to add targeting peptides to the surface of the protein cages. The RGD4C peptide was inserted into the AaLS sequence between positions 70 and 71, or the SP94 peptide was inserted into the C-terminus at the outer surface of AaLS. The RGD4C peptide specifically binds to the integrin avb3 receptor, which is overexpressed on the surface of endothelial cells in tumor vessels, and the SP94 peptide binds to the surface receptor on hepatocellular carcinoma cells. Fluorescent proteins can be linked to RGD4C-AaLS and SP94-AaLS to create diagnostic cancer probes. Bortezomib (BTZ) was linked to SP94-AaLS to effectively inhibit proteasome activity in hepatocellular carcinoma cells. AlDox, which can be released when pH is 5.5, was linked to RGD4C-AaLS to have a high cytotoxic effect on cancer cells that express high levels of the integrin [11]. The SP94 peptide is inserted into the encapsulin protein cage nanoparticle from *Thermotoga maritima*, which can also be targeted to liver cancer cells for cancer diagnosis or treatment [12]. Dox and curcumin can be released from the protein cage NPs in an acid-responsive manner with a pH range of 5.0–6.0. Targeting peptides and anticancer medications have recently been coupled to AaLS protein cages by the ST/SC creation of heteropeptide covalent connections, according to a research team [25]. The TNF-related apoptosis-inducing ligand (TRAIL) and control of EGFR downstream signaling, together with the overexpression of the epidermal EGFR in some tumor cells, have substantial effects on inducing apoptosis in tumor cells. GRP78 is an SP94 peptide receptor that was created on the surface of Pyrococcus furiosus ferritin Fn by genetic engineering. To create a nano-drug delivery system that targets hepatocellular carcinoma cells, the doxorubicin (Dox) was encapsulated into the cage using urea-controlled disassembly and reassembly of hccFn [38].

Dox was encapsulated with HFn, which minimized drug leakage in healthy organs and showed that HFn-Dox had a higher Dox concentration in mice tumors than free Dox administration [39]. This indicates that this protein cage NPs drug delivery method has a good safety profile. The primary treatment for breast cancer has been cytotoxic chemotherapy, and the use of protein cage NP vector delivery can successfully prevent cytotoxic drug damage to normal tissues [29]. Breast cancer cells often overexpress the surface receptors for the growth factors EGFR and HER2. Breast cancer cells are attracted to drug-carrying P22 VLPs, which can then be used to diagnose the disease by fusing fluorescent tags to EGFRAfb and HER2Afb and covalently binding them to the SP suggested by P22 VLPs. The cytotoxic protein small Singlet Oxygen Generator, an encapsulated protein cage that successfully targets breast cancer cells and causes their death, was loaded onto the surface of *T. maritima* encapsulin and fused to the genetically modified antibody protein DARPin9.29. Additionally, this team discovered that an 18.4 kDa protein can be fused directly to the surface of encapsulin without altering its stability or structure [53]. This discovery makes it easier to express some big proteins on the surface of encapsulin and opens up more application possibilities. Loading TNF-related apoptosis-inducing ligand (TRAIL) onto AaLS by covalent bonding and binding them to proapoptotic death receptors overexpressed in cancer cells is an effective protein therapy strategy for cancer [25].

The targeting effectiveness of protein cage NPs at tumor locations has recently received increased focus, and P22 VLPs modified with polyethylene glycol were chosen to investigate the targeting effectiveness in colorectal cancer models. In order to increase the duration of the drug’s circulation in vivo, polyethylene glycol on the surface of protein cage NPs is modified to keep it from being detected in blood vessels. The 7-day tumor model displayed the highest accumulation of P22 VLPs after 24 h of treatment compared to the 15-day and 21-day models in this investigation, and it was discovered that P22 VLPs accumulated mostly in tumor-associated macrophages, suggesting that P22 VLPs could be developed for the detection of early tumorigenic stages [54].

**Figure 4:**
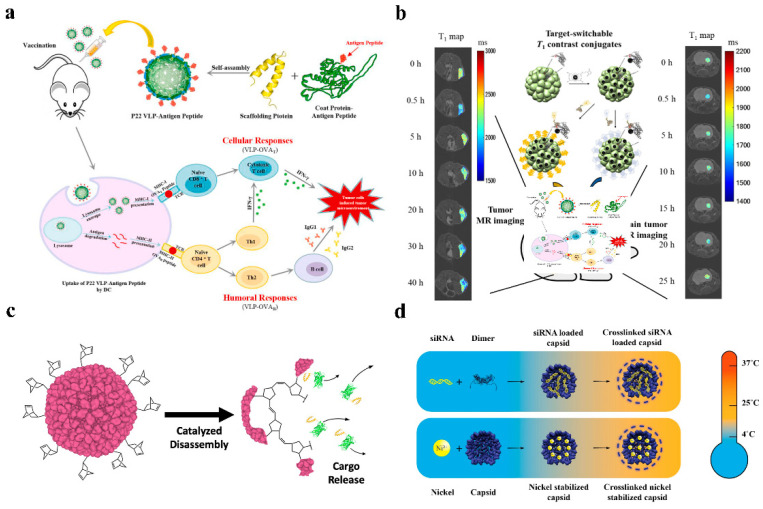
Application of several protein cage NPs. (**a**) P22 VLP-OVA capsid protein efficiently stimulated the CTL immune response in mice. Reprinted with permission from Ref. [55]. Copyright 2021, Elsevier Ltd. (**b**) Gd(III)-DOTA-AaLS for Nuclear Magnetic Resonance Imaging. Reprinted with permission from Ref. [26]. Copyright 2021, Elsevier B.V. (**c**) P22 VLPs encapsulates venom peptides and releases by ROMP disassembly strategy. Reprinted with permission from Ref. [64]. Copyright 2021, American Chemical Society. (**d**) Negatively charged siRNA is encapsulated by CCMV and cross-linked with DTSSP to provide stable VLPs for in vitro delivery. Reprinted with permission from Ref. [14]. Copyright 2019, American Chemical Society.

Cancer immunotherapy has also been practiced with P22 VLPs. The production of ovalbumin (OVA), OVA_B_, and OVA_T_ epitopes fused to the C-terminus of P22 VLPs capsid protein efficiently stimulated the cytotoxic lymphocyte (CTL) immune response in mice [55] (Figure 4a). The results of this work will serve as the basis for the creation of a preventive tumor vaccine.

### 3.2. siRNA Delivery

Negatively charged siRNA can be contained within CCMV due to the arginine structural domain’s facilitation of electrostatic interactions with negatively charged biomolecules. A protein cage NPs stable at 37 °C can be built for in vitro siRNA delivery using DTSSP crosslinker to couple lysine on the particle surface [14] (Figure 4d). Forkhead box transcription factor (FOXA1) was used as a therapeutic target in the application of CCMV delivery of siRNA, and cell penetrating peptides (CPPs), L17E, were covalently coupled to the surface of CCMV loaded with siRNA, effectively silencing FOXA1, the target gene of mcf-7 in breast cancer cells [56].

It was discovered that both CCMV and BMV VLPs could internalize into mcf-7 cells without targeting ligands and that CCMV could elicit an immunogenic response in raw264.7-blue macrophage cells, although BMV scarcely elicited one. Similar to CCMV, the plant virus bromemosaic virus (BMV) may effectively load siRNA, and the positively charged N-terminal portion of the BMV coat protein can be effectively loaded with siRNA [57]. It was possible to successfully release siRNA into tumor cells to silence genes by employing VLP-siGFP nanovectors, which had a mass ratio of 1:6 (siGFP/CP). As a result, BMV might be a better vector for siRNA delivery [56].

### 3.3. Peptide and Protein Delivery

Cancer, cardiovascular, and metabolic illnesses can all be effectively treated with peptide medications. The solubility, stability, and membrane permeability of peptide drugs, however, make them less desirable as carriers for the delivery of peptide drugs. Among these carriers, protein nanocages are excellent for the delivery of peptide drugs because they can successfully target and release peptide drugs to the focal site precisely. By way of illustration, the peptide drug for P22 VLPs were fused to the N-terminus of P22 CP and two different peptides, NuBCP-9 peptide and KLAK peptide, were linked by CTB cleavable linkers. The two peptides acted by different mechanisms and may have synergistic effects to kill tumor cells. Between the peptide and the CP as well as between the two peptides, CTB cleavable linkers are placed (Figure 4c). When this nano-drug delivery system is directed to the tumor site, the peptide medication is precisely released at the tumor site for tumor therapy in the presence of CTB, which is abundantly expressed in the tumor environment [47]. Enzymes like green fluorescent protein have been effectively put inside the protein shell by CCMV [34], P22 VLPs [51], and other protein cage NPs. The enzyme substrate can diffuse freely into the interior of the VLP through a 2 nm pore in the VLP coat. P22 VLPs have successfully encapsulated dehydrogenase D [58] within the protein cage and have been reported to be able to encapsulate fusion proteins up to 180 kDa in size without affecting the structure of the P22 VLPs [59].

### 3.4. Imaging

One of the most used techniques for clinical diagnosis is magnetic resonance imaging (MRI). Contrast agents (CA) are utilized to increase the resolution of MR images when it is insufficient, and both T1-weighted and T2-weighted (CAs) are frequently used [60].

The most widely used T1 CA are paramagnetic gadolinium (Gd(III)), but investigations have indicated that Gd is potentially cytotoxic and that using Gd-based contrast agents may result in systemic nephrotoxicity, necessitating complexation with chelating agents [26] (Figure 4b). Due to the protein cage NP’s distinctive spherical hollow form, biodegradability, simplicity of mutagenesis, and chemical coupling changes, it is possible to fix the contrast agent on the protein cage NP and reduce the release rate of the contrast agent. Recombinant human heavy chain ferritin with Gd tagging was designed to target T1 contrast agents (HFn-Gd) [4]. the cavity of the P22 “wiffleball” encapsulates the DTPA-Gd complex with a greater resolution than free DTPA-Gd, and with 1900 Gd^3+^ included in each P22 VLP [61]. Gadolinium-tetraazacyclododecane tetraacetic acid (GdDOTA) is attached to CCMV by reaction with lysine residues on the surface of the viral capsid [62]. The Gd(III)-DOTA complex was chemically attached to the surface of AaLS to create a targeted T1 contrast agent after a tumor-targeting peptide was changed on its surface using ST/SC to generate a covalent heteropeptide connection. The utilization of flavoprotein (AfFtnAA) nanocages with a slow iron release rate, high iron loading capacity, and thermal stability encapsulates magnetic nanostructures (MNS) based on superparamagnetic iron oxide and metal ferrite NPs to produce increased contrast in MR imaging [63].

## 4. Conclusions and Discussion

The study of nanomaterial drug delivery systems has attracted more attention recently, which has aided in the creation of protein cage nanomaterials. Protein cage nanomaterials offer new opportunities for developing sophisticated protein nanomaterial drug delivery systems because of their favorable biocompatibility, lack of toxicity, and ideal internal cavity shape, even on the surface of the protein cage NPs, for drug delivery. Numerous studies have also demonstrated that protein cage NPs can effectively protect protease activity and that, in contrast to free enzymes, enzymes encapsulated in protein cage NPs can maintain enzyme activity at high concentrations, with drug protein concentrations occasionally reaching 300 mg/mL [42,65]. This work comprehensively describes the primary ways for encapsulating pharmaceuticals in low-cost and easily accessible protein cage NPs, how they are released, and their prospective applications for the biomedical field (Table 2), such as applications in the delivery of antitumor drugs and vaccines, siRNA, peptides and protein-based drugs, and applications in Biologic imaging techniques.

This work enumerates the protein cage NPs produced by eukaryotic or prokaryotic expression systems. The majority of these NPs are produced in large quantities by *E. coli* expression purification, which is easy to use and inexpensive to run. As a result, many drug carriers with high purity can be produced, and even protein cage NPs for encapsulating drugs can be produced directly, minimizing the loss of carriers and drugs during the encapsulation process. Protein cage NPs are convenient for targeting modification. Targeting drug-loaded protein cage NPs to a specific location before releasing the drug can effectively improve drug utilization and even prevent the release of some toxic drugs during delivery, reducing the harm the drug causes to healthy tissues and cells. For a variety of drug delivery applications, including targeted tumor cell treatment, contrast, siRNA, and peptides, protein cage NPs hold great promise. Although VLPs have shown great efficacy, there are still limitations to their clinical use as drug delivery tools. HFn nanocages are naturally present in humans and do not elicit inflammatory or immune responses [36]; however, most Protein cage NPs, like other NPs, are cleared by phagocytic and dendritic cell-mediated clearance. The CD40L/CD47 Self-peptide can bind to SIRP-α and inhibit macrophage phagocytosis [66,67], the tobacco mosaic virus’s immunogenicity has been decreased using serum albumin (SA) [56], and PEGylation can shield the surface of Protein cage NPs, allowing NPs to go undetected in the vasculature, prolonging body circulation, and inhibiting macrophage phagocytosis, but it is also a major challenge in application [54,68,69].

Protein cage NPs are used to encapsulate peptides and protein-based drugs, some enzymes involved in biosynthesis, and multi-enzyme cascade reactions for cancer therapy. Protein cage NPs can be modified both internally and externally to mimic the cellular environment, particularly large inner diameter protein cage NPs such as P22 VLPs, developed as biosynthetically active nanoreactors. With the help of further in-depth research on the safety, stability, toxicity, and pharmacokinetics of novel protein cage NPs, we hope that they will be successfully tested in preclinical and clinical settings. We also hope that this review will give researchers new ideas and insights for developing novel protein cage NPs for drug delivery.

**Table 2:** Protein cage NPs for drug encapsulation and delivery.

Protein Cage Nps Type	Cargo	Encapsulation Method	Surface Modification	Release Method	Application	References
P22	Alexa Fluor 647	Covalent modification	polyethylene glycol 1000 (PEG1K)	-	Anti-tumor (targeting efficiency)	[54]
	CD47 peptide/CD40L	Covalent modification	decoration protein	-	None	[66]
	AlDo	Covalent modification	S133C or K118C EGFRAfb and HER2Afb targeting peptides	pH = 5.0	Anti-tumor (human breast cancer cells)	[29]
	HA_head_	Covalent modification	None	Antigen-antibody reaction	IAV vaccine	[32]
	NuBCP-9 peptide and KLAK peptide	Fusion expression	None	Cathepsin B cleavage	Peptide and protein delivery Anti-tumor	[47]
	GCL/GshF	Fusion expression	None	Enzyme activity	GSH-deficient	[70]
	Cytochrome P450 enzymes/Protoporphyrin IX	Fusion expression	PEG (EST)	Enzyme prodrug therapy	Anti-tumor (breast tumor cells)	[71]
	DTPA−Gd	Covalent modification	K118C	As contrast agents	Imaging	[61]
Fn	Dox/Camptothecin	Diffusion	hydrophobic peptides at the C-terminus of HFn	pH = 5.0	Anti-tumor (human glioblastoma cells)	[6]
	Dox	Diffusion	engineering 4-fold channel-nicked HFn	Acid-responsive	Anti-tumor (cervical cancer cells and breast tumor cells)	[18]
Fn	GFP(+36)	Electrostatic adsorption	AfFtn WT/K150A, R151A	-	None	[21]
	SOD	Covalent modification	tF1iC (E131C)	Enzyme activity	Anti-inflammatory effect	[28]
	Dox	Disassembly/reassembly(pH)	None	-	None	[5]
	Dox	Disassembly/reassembly(Urea)	SP94 targeting peptides	pH = 5.0	Anti-tumor (hepatocellular carcinoma)	[38]
	Dox	Disassembly/reassembly(Urea)	None	pH = 5.0	Anti-tumor (human colon cancer cells)	[39]
	Curcumin	Disassembly/reassembly(Urea)	None	Acid-responsive	Anti-tumor (breast cancer cells)	[35]
	multivalent microplasmin	Fusion expression	Fibrin clot-targeting peptides	Enzyme activity	Anti-tumor	[49]
	Gd-DTPA	Covalent modification	None	As contrast agents	Imaging (breast cancer and pancreatic cancer)	[4]
	Fe	Covalent modification	AfFtn K150A/R151A	As contrast agents	Imaging (macrophage cell)	[63]
AaLS	Gd(III)-DOTA	Covalent modification	HER2 or EGFR targeting peptides	As contrast agents	Imaging (tumor cells)	[26]
	Sec	Covalent modification	C37A/E122C	Glutathione or dithiothreitol.	Anti-tumor (human cancer cell lines)	[27]
	AlDox	Covalent modification	R108C RGD4C and SP94 targeting peptides	pH = 5.5	Anti-tumor (hepatocellular carcinoma)	[11]
	TRAIL	Covalent modification	R108C EGFRAfb targeting peptides	Ligand-receptor interaction	Anti-tumor (human epidermoid cancer cell)	[25]
	Periostin peptide	Fusion expression	None	Ligand-receptor interaction	Pro-angiogenic activity (Peripheral artery disease)	[50]
CCMV	siRNA	Electrostatic adsorption	DTSSP cross-linker	Transfection	siRNA Delivery	[14]
	siRNA	Disassembly/reassembly(pH/salt)	M-lycotoxin peptide L17E	Transfection	siRNA Delivery	[56]
Encapsulin	GCaMP	Fusion expres-sion	Amino acid changes and deletions in pore-forming loop region	-	None	[19]
	AlDox	Covalent modification	123C SP94 targeting peptides	pH = 5.5	Anti-tumor Imaging (hepatocellular carcinoma)	[12]

## Data Availability

Not applicable.
